# Supplementary material for: A powerful and efficient set test for genetic markers that handles confounders
Source: Bioinformatics. 2013 Apr 18;29(12):1526–33. doi: 10.1093/bioinformatics/btt177 (PMC3673214; doi:10.1093/bioinformatics/btt177)
Supplement: Supplementary Data [file supp_29_12_1526__index.html]

A powerful and efficient set test for genetic markers that handles confounders — A powerful and efficient set test for genetic markers that handles confounders — Supplementary Data 

# A powerful and efficient set test for genetic markers that handles confounders

## Supplementary Data

files

**Files in this Data Supplement:**

- Supplementary Data - xlsx file
